# A lack of association between vitamin d-binding protein and 25-hydroxyvitamin d concentrations in pediatric type 1 diabetes without microalbuminuria

**DOI:** 10.1186/1687-9856-2015-S1-P16

**Published:** 2015-04-28

**Authors:** Hwa Young Kim, Young Ah Lee, Hae Woon Jung, So Youn Kim, Kyung A Jeong, Keun Hee Choi, Jieun Lee, Ju Young Yoon, Sei Won Yang, Choong Ho Shin

**Affiliations:** 1 Department of Pediatrics, Seoul National University College of Medicine, Seoul, Korea; 2 Department of Pediatrics, Sungkyunkwan University College of Medicine, Samsung Medical Center, Seoul, Korea; 3 Center for Pediatric Oncology, National Cancer Center, Goyang, Korea

## 

The risk of vitamin D deficiency might increase along with the increased urinary loss of vitamin D binding protein (VDBP) consequent to impaired 25-hydroxyvitaminD (25-OHD) circulation. We aimed to evaluate the possible increased urinary loss of VDBP, a correlation between VDBP and circulating 25-OHD levels, and the risk factors influencing low vitamin D levels in pediatric type 1 diabetes patients without microalbuminuria.

Subjects with T1DM without microalbuminuria (n=45) and age-matched healthy control subjects (n=29), aged 9–14 yr, residing in Seoul and the Gyeonggi-Do in Korea (37°N) were studied. Height, weight and pubertal stage were evaluated. The percentage of body fat was measured by bioelectrical impedance analysis (inbody). A questionnaire was used to assess the amount of daylight outdoor activity and vitamin D intake. Serum levels of calcium, phosphorus, intact parathyroid hormone, 25-OHD, 1,25 dihydroxyvitamin D and VDBP, as well as urinary levels of VDBP, microalbumin and creatinine (Cr) were measured. 25-OHD deficiency was defined as ≤20 ng/mL.

The urinary VDBP to Cr ratio (VDBPCR) in type 1 diabetes patients was higher than that in the control group (P = 0.016) and correlated positively with the urinary microalbumin to Cr ratio in both groups (P <0.001). The serum 25-OHD levels did not correlate with the serum VDBP or urinary VDBPCR. A multivariate regression analysis including known vitamin D deficiency risk factors (age, gender, body fat percentage, vitamin D intake, daylight outdoor hours, and urinary VDBPCR) revealed that daylight outdoor hours (β = 2.881, P = 0.009) and vitamin D intake (β = 2.342, P = 0.050) affected the 25-OHD levels in type 1 diabetes patients.

In pediatric type 1 diabetes patients without microalbuminuria, the urinary VDBP loss was increased proportionally with the urinary mACR. However, the urinary VDBPCR did not correlate with the serum 25-OHD levels. The factors associated with 25-OHD levels during winter periods were daylight outdoor hours and vitamin D intake.

